# Differential effects of excess high-fructose corn syrup on the DNA methylation of hippocampal neurotrophic factor in childhood and adolescence

**DOI:** 10.1371/journal.pone.0270144

**Published:** 2022-06-17

**Authors:** Itsuki Kageyama, Hiroya Yamada, Eiji Munetsuna, Mirai Yamazaki, Yoshitaka Ando, Genki Mizuno, Ryosuke Fujii, Yuki Nouchi, Takuya Wakasugi, Tomohide Sakakibara, Atsushi Teshigawara, Hiroaki Ishikawa, Yohei Shimono, Koji Suzuki, Shuji Hashimoto, Koji Ohashi

**Affiliations:** 1 Department of Informative Clinical Medicine, Fujita Health University School of Medical Sciences, Toyoake, Aichi, Japan; 2 Department of Preventive Medical Sciences, Fujita Health University School of Medical Sciences, Toyoake, Aichi, Japan; 3 Department of Hygiene, Fujita Health University School of Medicine, Toyoake, Aichi, Japan; 4 Department of Biochemistry, Fujita Health University School of Medicine, Toyoake, Aichi, Japan; 5 Department of Medical Technology, Kagawa Prefectural University of Health Sciences, Takamatsu, Kagawa, Japan; University of Louisville, UNITED STATES

## Abstract

Consumption of fructose-containing beverages such as high-fructose corn syrup (HFCS) is increasing, raising concerns about the negative effects of excessive fructose intake. A recent report indicated that excess HFCS intake impairs hippocampal function. In this study, we focused on neurotrophic factors (NFs) in the hippocampus from the viewpoint of epigenetics to clarify the adverse effects of fructose. We analyzed the effects of HFCS intake on hippocampal function in three age categories: childhood and adolescence (postnatal day (PD) 21–60), young adulthood (PD60-100), and late adulthood (PD100-140). For the experiments, male Sprague-Dawley rats were divided into three age categories, the control group was received distilled water and the HFCS group was received 20% HFCS solution for 40 days in each period. We analyzed mRNA and protein levels for qPCR and western blotting, respectively, of a hippocampal NF, brain-derived neurotrophic factor (Bdnf). HFCS consumption reduced hippocampal *Bdnf* mRNA and protein expressions in childhood and adolescence. Moreover, pyrosequencing assays revealed increased DNA methylation at the *Bdnf* promoter in childhood and adolescence. This Bdnf levels reduction may be due to hypermethylation of the promoter regions. It should be noted that this phenomenon was observed only in childhood and adolescence fructose consumption. Our results indicate that the sensitivity of the hippocampus to fructose may vary with age. This study provides insight into the adverse effects of excessive HFCS consumption on the hippocampus in children.

## Introduction

A diet high in added sugars, especially fructose, has adverse metabolic outcomes and is associated with metabolic disorders [1]. Fructose is a natural sugar found in fruits and vegetables often consumed in the form of high-fructose corn syrup (HFCS). HFCS, which consists of 45% glucose and 55% fructose, is low in cost and high in sweetness; hence, its consumption has been increasing over the last 50 years [2]. According to various studies, increased HFCS consumption is considered a risk factor for metabolic syndrome, dyslipidemia, insulin resistance, obesity, and type 2 diabetes [1, 3–7]. Therefore, the social concern about excessive fructose consumption has been growing in recent years.

Emerging data also indicate that high-fructose diets have a marked impact on brain function [8, 9], particularly within the hippocampus, a brain region involved in learning, memory, and food intake regulation that is particularly susceptible to metabolic impairment [10–12].

Excessive consumption of fructose-containing beverages such as HFCS, a hallmark of modern lifestyles, is especially pronounced in the young population [13]. Childhood is a critical period for the maturation of the hippocampal circuitry [14] and heightened neural plasticity [15, 16]. In addition, it is an important period of susceptibility to various stresses with long-term consequences in the cognitive and memory domains. As metabolism and physiological functions differ greatly between childhood and adulthood, it is expected that susceptibility to the adverse effects of fructose may also differ between childhood and adulthood. However, only a few studies have focused on the duration of intake while examining the adverse effects of fructose. Hsu et al. showed that the effects of high fructose diets on hippocampal function are particularly damaging during sensitive periods of neurocognitive development, such as childhood and adolescence [17]. Recently, Clark et al. demonstrated that higher dietary intake of added sugars, particularly fructose, was associated with alterations in hippocampal structure and connectivity during childhood in 103 children aged 7–11 years [18].

NFs are composed of four types of liquid factors: nerve growth factor (Ngf), Bdnf, neurotrophin-3 (Nt3), and neurotrophin-4 (Nt4) [19]. NFs play an important role in the development of brain functions during growth [11, 20, 21]. NF deficits have also been shown to hamper cognitive processes through the inhibition of crucial mechanisms such as long-term potentiation [22]. Furthermore, Bdnf and Ngf may play vital roles in mediating processes that associate adulthood environment with brain development and behavior [23]. Based on previous findings, excess fructose intake causes cognitive decline and impaired neurogenesis and might lead to aberrant expression of these NFs.

In this study, we focused on NFs in the hippocampus from the viewpoint of epigenetics to clarify the adverse effects of fructose. Furthermore, we aimed to clarify the effects of HFCS intake on hippocampal function in three age categories: childhood and adolescence (PD21-60), young adulthood (PD60-100), and late adulthood (PD100-140).

## Materials and methods

### Ethics statement

The study protocol was approved by the Animal Ethics Committee of Fujita Health University (Permit No. H0862) and complied with guidelines of the National Research Council’s Guide for the Care and Use of Laboratory Animals.

### Animal experiments

All animals were housed in an environmentally controlled cage at room temperature (23 ± 3°C) under a 12:12 h light-dark cycle. The experimental period was divided into three parts: childhood and adolescence (21–60 days old: Period I), young adulthood (60–100 days old: Period II), and late adulthood (100–140 days old: Period III). 21, 60, 100 days old male Sprague-Dawley rats (Shizuoka Laboratory Center, Shizuoka, Japan, RRID: RGD_2314928) were divided into control (C, n = 7–8) and HFCS groups (H, n = 7–8) in each period, and fed ad libitum with distilled water or 20% HFCS water in separate water bottle. All animals were housed in groups of 2–3 and given 40-days ad libitum access to their respective water source and standard chow (Nisshin Seifun Group, Tokyo, Japan) during the experimental period. 20% HFCS solution was prepared using 75% HFCS (Japan Corn Starch, Tokyo, Japan) and distilled water. Body weight was measured once per week. Finally, access to food was withdrawn from all animals 6 h before dissection.

The rats were sacrificed following isofluorane. A surgical plane of anesthesia was confirmed when a toe pinch failed to elicit a change in respiratory rate or pattern [24].

### Hippocampal DNA and RNA extraction

Hippocampal tissues of all animals were dissected to extract DNA and RNA from the hippocampus. To extract DNA, we used NucleoSpin Tissue (Takara, Otsu, Japan). Total RNA was isolated from the hippocampal tissues using TRIzol reagent (Thermo Fisher Scientific, Waltham, MA, USA). Nucleic acid quality was confirmed by the 260/280 (>1.8) and 260/230 (>1.8) optical density ratio by using a NanoDrop ND-1000 spectrophotometer (Thermo Fisher Scientific). Samples were stored at -80°C for quantitative analysis of DNA methylation and quantitative assessment of mRNA expression.

### Quantification of mRNA expression

As in our previous report [25] qPCR for quantitative assessment of mRNA expression was performed using the THUNDERBIRD SYBR qPCR Mix (Toyobo, Osaka, Japan) and QuantStudio 7 Flex system (Thermo Fisher Scientific) at 95°C for 1 min, followed by 40 cycles of 95°C for 15 s, 55°C for 30 s, and 60°C for 1 min. The expression levels of the target genes were normalized using beta-actin (Actb) mRNA as an internal control. We compared the C group with the H group by fold change using the 2^− ΔΔct^ method [26, 27]. To analyze the specificity of the qPCR, a melt-curve analysis was subsequently performed as follows at 95°C for 1 min, 55°C for 1 min, then the temperature was increased by 0·5°C every 10 s from 55 to 95°C. The primers used to amplify the target genes were as follows: Ngf: forward: 5′-CAGAGTTTTGGCCTGTGGTC-3′, reverse: 5′-GGACATTACGCTATGCACCT-3′; Bdnf: forward: 5′-CTCCGCCATGCAATTTCCAC-3′, reverse: 5′-CAGCCTTCATGCAACCGAAG-3′; Nt3: forward: 5′-ACGGCAACAGAGACGCTAC-3′, reverse: 5′-CTCCAAAGGGGTGCTGTC-3′; Nt4: forward: 5′-GCCCCAGAGTGAGGAGGT-3′, reverse: 5′-GGAGGAGGAGAAGGAAAAGG-3′; Actb: forward: 5′-CCCGCGAGTACAACCTTCT-3′, reverse: 5′-CGTCATCCATGGCGAACT-3′.

### Western blot analysis

Hippocampal tissues were homogenized in RIPA buffer (Wako Pure Chemicals, Osaka, Japan). The total protein concentration was determined using the BCA Protein Assay Kit (Thermo Fisher Scientific). As previously reported [28, 29], SDS-PAGE and western blotting were carried out as follows: the proteins were boiled in EzApply Buffer (Atto, Tokyo, Japan), run in SDS-PAGE gels and transferred to PVDF membranes (Atto, Tokyo, Japan). The membranes were then incubated overnight at 4°C with primary antibodies against Bdnf (1:1000; Santa Cruz Biotechnology Cat# sc-546, RRID: AB_630940), Actb (1:2000; Abcam Cat# ab8227, RRID: AB_2305186), total Akt (1:1000; Cell Signaling Technology Cat# 9272, RRID: AB_329827), and phosphorylated Akt (1:1000; Cell Signaling Technology Cat# 9271, RRID: AB_329825). Subsequently, the membranes were incubated with a secondary antibody conjugated with horseradish peroxidase for 1 h at room temperature, and the immunoreactive bands were visualized using ECL+ chemiluminescence detection reagent (GE Healthcare, Amersham, UK). The intensities of the chemiluminescence of specific bands were analyzed using a FUSION—Chemiluminescence Imaging System (M&S Instruments, Osaka, Japan).

### Analysis of CpG methylation of hippocampal *Bdnf*

Bisulfite pyrosequencing was performed to determine the proportion of methylated CpG site, as in our previous report [30, 31]. First, we treated DNA (250 ng) with bisulfite using the EpiTect Fast DNA Bisulfite Kit (Qiagen, Hilden, Germany) to convert unmethylated cytosines to uracils while leaving methylated cytosines unmodified. PCR was then performed using bisulfite-treated DNA (10 ng/μL) with the TaKaRa EpiTaq HS (for bisulfate-treated DNA; Takara) in a total volume of 20 μL. Samples were washed in PyroMark Q24 Vacuum Workstation (Qiagen) with 70% ethanol and PyroMark Denaturation Solution (Qiagen) to separate the complementary strand from the biotin-tagged strand. The samples were then washed with PyroMark Washing Buffer (Qiagen). Samples were then annealed with sequencing primers in PyroMark Q24 Plate (Qiagen), followed by heating at 80° for 5 min using a preheated PyroMark Q24 plate holder. The heated plate containing single-stranded template DNA and sequencing primers was transferred to the PyroMark Q24 Instrument to begin the run to collect methylation data. Finally, site-specific methylation rates were collected visually using PyroMark Q24 Advanced software and data quality was assessed according to the manufacturer’s guidelines. We used the following PCR primer sequences for the PyroMark Assay Design SW 2.0 (Qiagen): *Bdnf*: forward: 5′-AGGTAGAGGAGGTATTATATGATAG-3′; reverse: 5′-biotin-ATTTCCCCTTCTCTTCAATTAAA-3′, and sequencing: 5′-AGGTAGAGGAGGTATTATAT-3′.

### Statistical analysis

Statistical difference of the results was tested using 2-tailed Student t test or one-way ANOVA. Data are expressed as mean ± standard deviation (SD). Values of p < 0.05 were considered statistically significant.

## Results

### Effect of HFCS consumption on body weight

**Fig 1:**
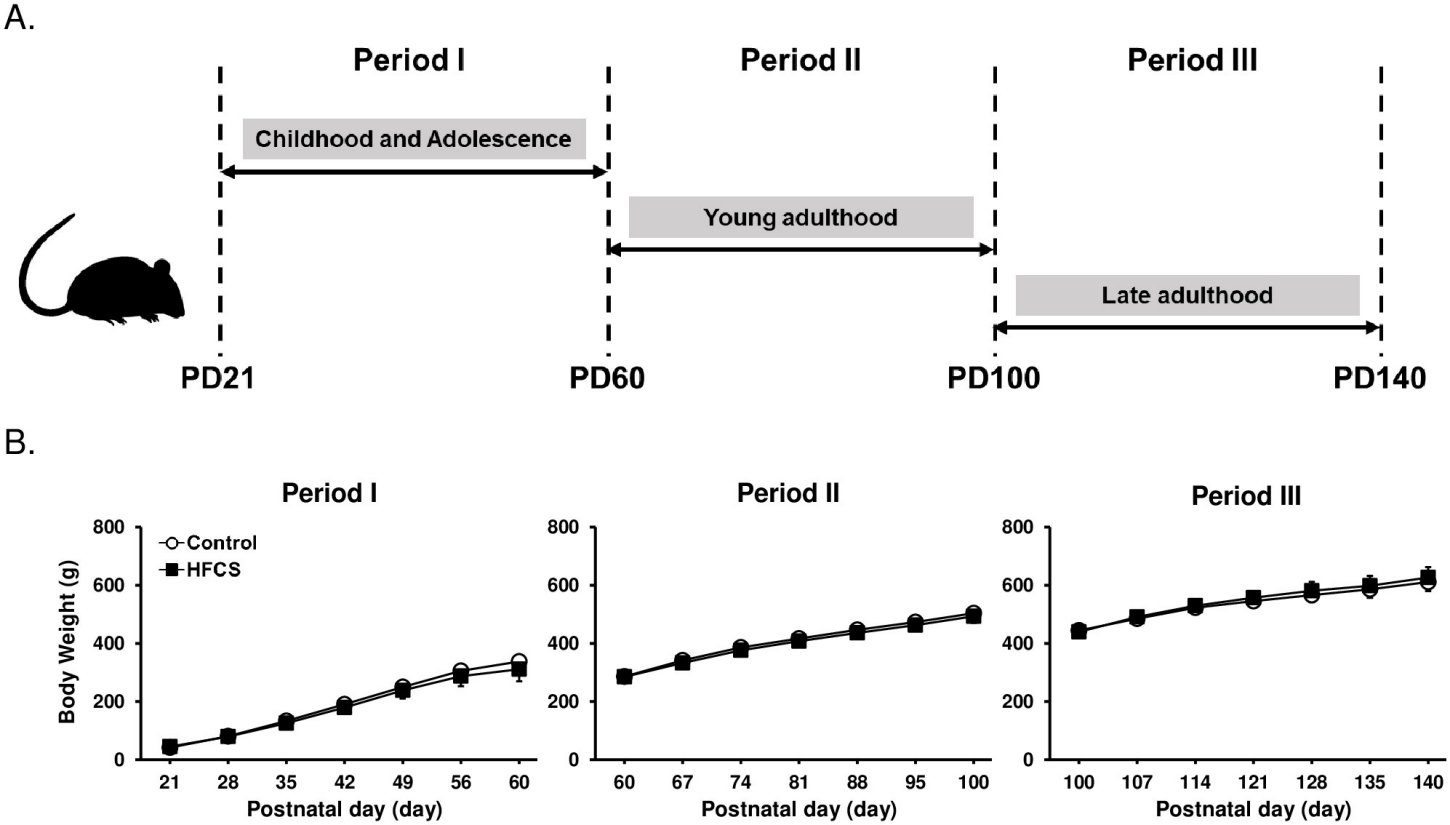
Body weight during each experimental period. (A) Experimental schedule of this study. (B) No significant differences were observed in the body weight between the two groups. Period I: C and H, n = 7, Period II: C and H, n = 8, Period III: C and H, n = 7. C, Control group; H, HFCS group. Data are presented as the mean ± SD.

The model used in this study is shown in Fig 1A. We evaluated the effect of HFCS intake on body weight gain during each period (Fig 1B). The body weight showed a group x days interaction (Period I: F(6, 66) = 1.96, Period II: F(6, 84) = 1.30, Period III: F(6, 72) = 1.663, p > 0.05 for each). No significant differences were observed in body weights between group.

### HFCS intake influences the hippocampal expression of Bdnf in Period I

**Fig 2:**
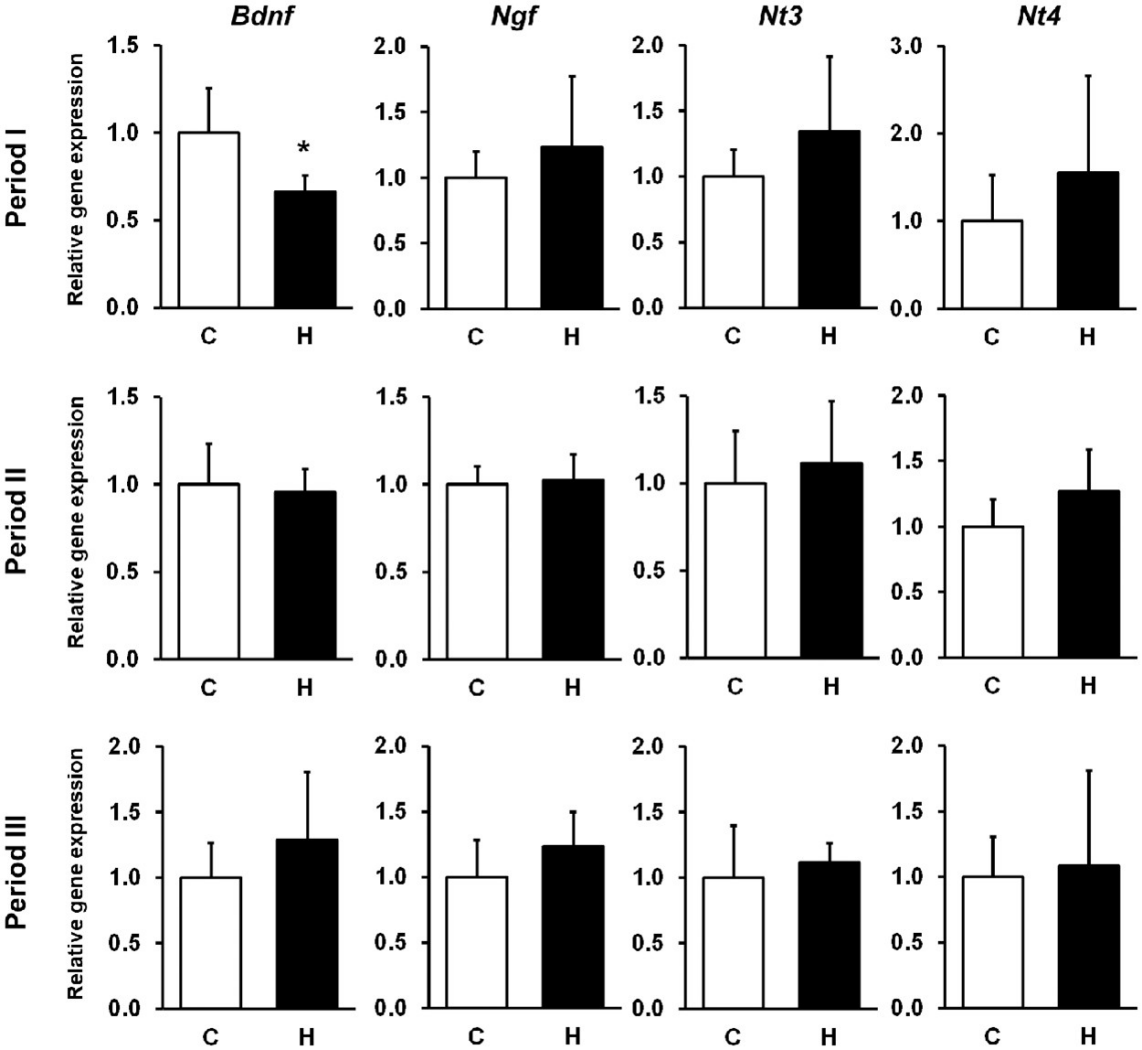
Hippocampal mRNA levels of NFs. Each mRNA was quantified by qPCR. The results are expressed as the ratio of the relative intensity of levels of gene expression to *Actb*. *Bdnf* gene expression was reduced in the Period I with HFCS. Period I: C, n = 5; H, n = 6, Period II: C, n = 8; H, n = 7, Period III: C, n = 7; H, n = 6. C, Control group; H, HFCS group; data are presented as mean ± SD.*p < 0.05.

**Fig 3:**
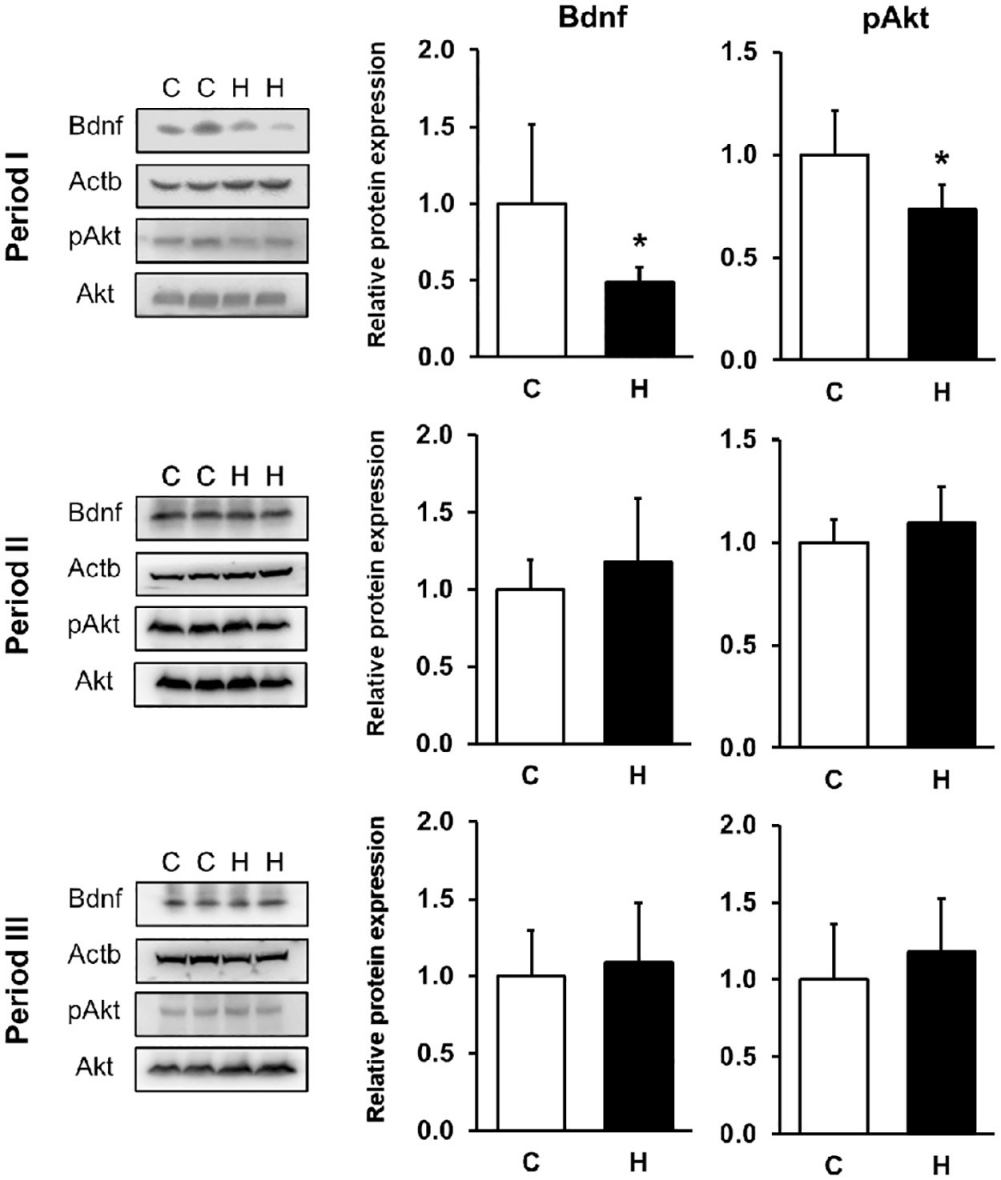
Hippocampal Bdnf and pAkt protein expression. The results are expressed as the ratio of relative intensity of protein expression levels to Actb or Akt. Hippocampal Bdnf and pAkt protein levels were decreased in the Period I with HFCS. Period I: C, n = 5; H, n = 6 (Bdnf), C and H, n = 6 (pAkt), Period II: C and H, n = 8 (Bdnf), C and H, n = 8 (pAkt), Period III: C and H, n = 7 (Bdnf), C and H, n = 6 (pAkt). C, Control group; H, HFCS group; data are presented as mean ± SD.*p < 0.05.

To confirm that the consumption of HFCS induces effects on hippocampal gene expression related to brain development, we quantified the mRNA levels of NFs. *Bdnf* gene expression was measured by targeting *Bdnf* exon IV, which is involved in neural activity-dependent *Bdnf* expression in rodents [30]. Hippocampal *Bdnf* gene expression was reduced in Period I (C group: 1.0 ± 0.3; H group: 0.7 ± 0.1; p < 0.05) (Fig 2). We also found the level of Bdnf protein was reduced significantly (C group: 1.0 ± 0.5; H group: 0.5± 0.1; p < 0.05) (Fig 3). As expected, hippocampal pAkt expression, a downstream target of Bdnf signaling, was reduced in Period I (C group: 1.0 ± 0.2; H group: 0.7± 0.1; p < 0.05) (Fig 3).

### Response to HFCS exposure in hippocampal *Bdnf* methylation levels at promoter region

**Fig 4:**
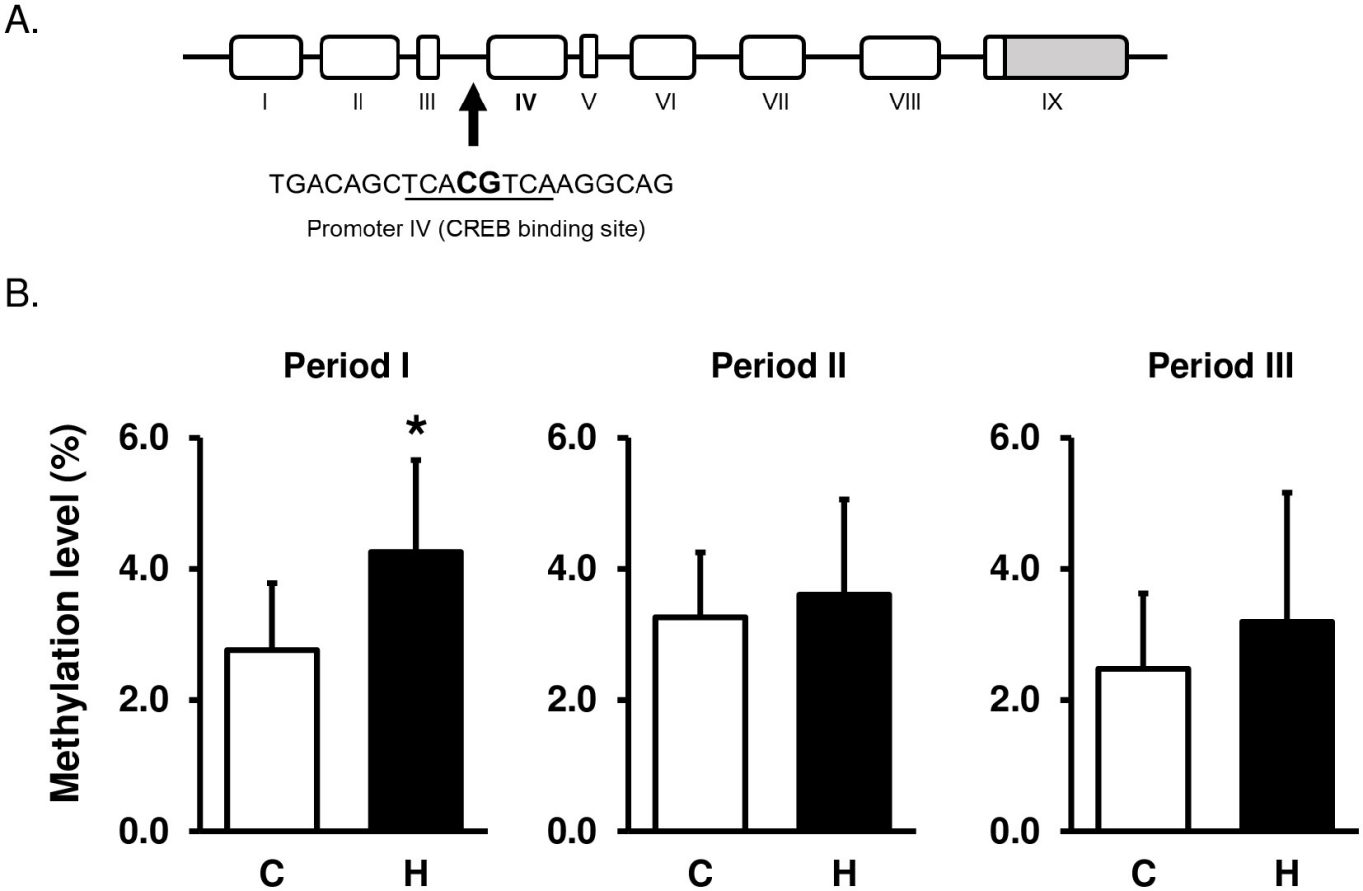
Hippocampal DNA methylation levels of the *Bdnf* promoter IV. (A) The schematic shows the target sequence of Bdnf exon IV promoter for pyrosequencing analysis. (B) HFCS intake induced high methylation levels of *Bdnf* promoter regions at the CREB binding site in Period I. Period I: C, n = 7; H, n = 6, Period II: C, n = 5; H, n = 6, Period III: C and H, n = 7. C, Control group; H, HFCS group. Data are presented as the mean ± SD.*p < 0.05.

We analyzed the CpG methylation levels at the CREB binding site in the targeted exon IV promoter of *Bdnf* using the bisulfite pyrosequencing method. HFCS consumption increased methylation level in the promoter of *Bdnf* during Period I (C group: 2.8 ± 1.0%; H group: 4.3 ± 1.4%; p < 0.05) (Fig 4B). There were no significant changes during the other periods. These results suggest that excess HFCS consumption in Period I induces high methylation of the *Bdnf* promoter region, which may lead to the suppression of gene expression (Figs 2 and 4).

## Discussion

**Fig 5:**
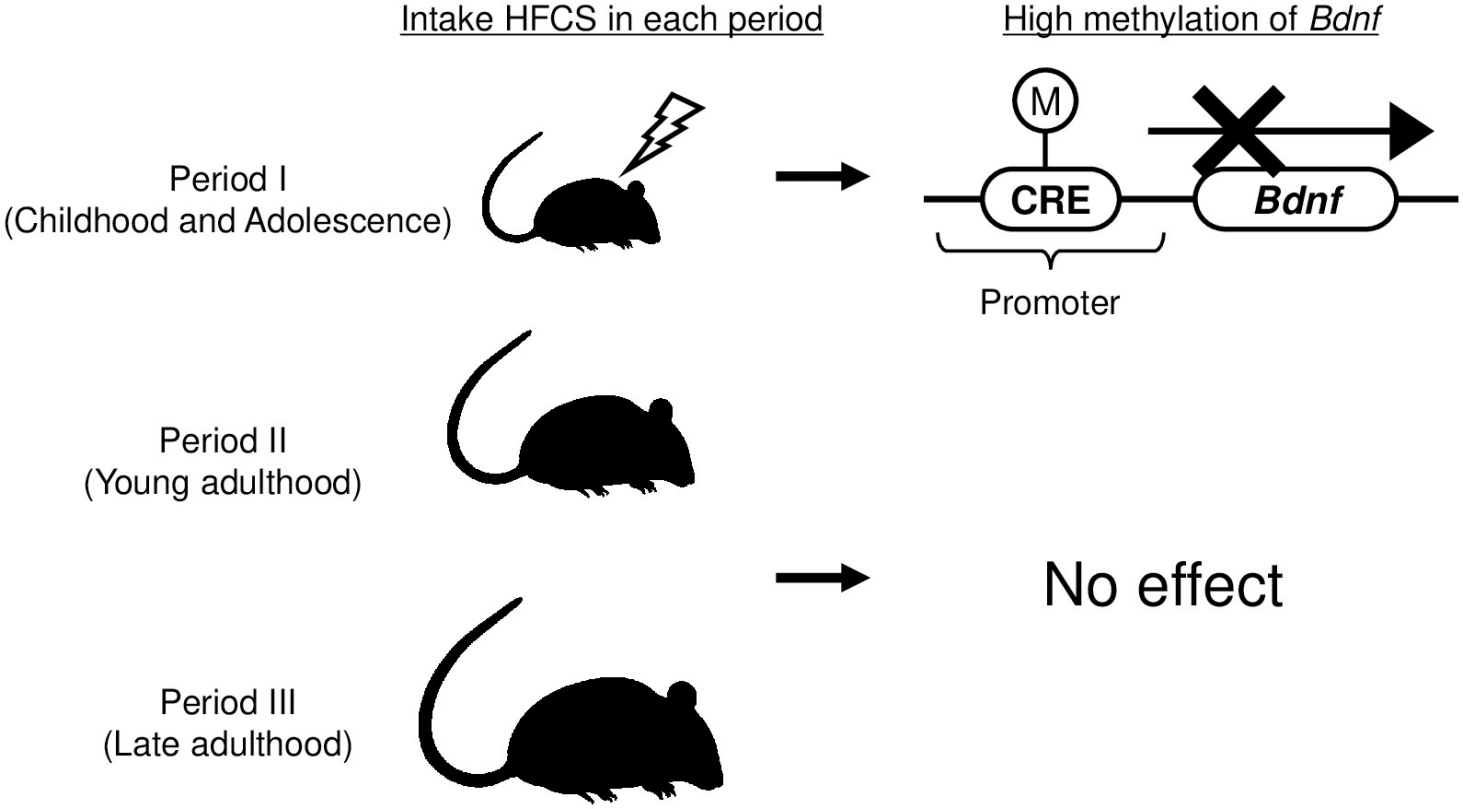
Differences in sensitivity to HFCS consumption in Period I. Excessive HFCS intake induces aberrant DNA methylation of *Bdnf* promoters in Period I but has no effect on the other periods. This specific change in Period I might cause the downregulation of Bdnf expression and finally hippocampal dysfunction.

In this study, we evaluated the effects of excess HFCS solution intake on the DNA methylation status of NFs in the hippocampus. The results showed that only in Period I, a decrease in the hippocampal *Bdnf* gene expression and Bdnf protein levels was found through increase in DNA methylation (Fig 5).

Recently, several studies showed that the expression of neurotrophic factors is regulated by epigenetic modifications, such as DNA methylation [11, 32, 33]. DNA methylation plays an important role in a variety of biological processes. This regulation is known to occur in the CpG site, where cytosine and guanine are linked, and has been reported to vary with environmental factors [10]. We previously indicated the adverse effects of chronic fructose intake on the DNA methylation levels of hepatic PPARα and CPT1A promoters, and on mitochondrial DNA in adult rats [3, 7]. These findings clearly indicate that epigenetic modifications, especially DNA methylation, are highly sensitive to high levels of fructose consumption. DNA methylation is also highly sensitive to excessive stress and poor nutritional status in childhood [12, 34].

There are some reports that the hippocampus is particularly susceptible to metabolic impairment [10–12]. Epidemiological studies have reported an association between increased fructose intake and cognitive dysfunction such as dementia [35]. Experimental animal studies have shown that high fructose consumption leads to a reduction in hippocampal neurogenesis [36] and altered mitochondrial activity [37] suggesting a potential mechanistic basis for fructose-induced cognitive deficits [17, 38]. In addition, a high-fructose diet is associated with neuroinflammation [17] and a high level of oxidative stress [39], both of which are implicated as risk factors in the pathogenesis of neurodegenerative disorders [40].

Several studies have shown that fructose intake has negative effects on the hippocampus. For example, short-term fructose intake without metabolic syndrome has been reported to decrease hippocampal weight and the number of neurons [41]. It has also been reported that fructose water causes inflammation in the hippocampus [42]. Thus, the adverse effects of fructose on the hippocampus are gradually becoming clearer, but the relationship between the sensitivity of the hippocampus to fructose and age is still unknown. To the best of our knowledge, the only report is that of Cigliano et al. They reported that when fructose was fed to young and adult rats, hippocampal inflammation and oxidative stress were observed in both age groups [39], indicating that age-related changes in fructose sensitivity are similar across age groups. In contrast, the present study found that hippocampal Bdnf expression was differentially sensitive to fructose with age (Fig 5). Interestingly, only Bdnf expression in Period I was reduced by fructose ingestion. Given the fact that childhood is a particularly important period for hippocampal development, fructose-induced hippocampal dysfunction may have a significant impact on tissue disorders. Bdnf is known to play a role in hippocampal neurogenesis, neuroprotection, maintenance of synaptic plasticity, and memory formation [20, 43–45]. Bdnf has also been associated with several diseases. It has been reported that Bdnf expression in the brain is decreased in patients with Alzheimer’s disease [46, 47]. Decreased hippocampal Bdnf expression leads to decreased neurogenesis and cognitive function [48, 49]. Collectively, the effects of reduced hippocampal Bdnf expression in Period I may persist throughout life.

Furthermore, once methylation modifications are introduced, they are maintained for a long time. Therefore, a decrease in Bdnf signaling is expected to occur over a relatively long period of time. This idea is supported by our previous report that methylation modification of the B*dnf* promoter in 21-day-old hippocampi was maintained for at least 2 months [30]. Thus, the adverse effects of fructose ingestion during puberty may be long-lasting. We conclude that fructose intake in childhood and adolescence is more harmful to hippocampal function than fructose intake in adulthood.

Several studies have reported that reduced Bdnf expression is associated with hippocampus-dependent cognitive decline [50, 51]. Heldt SA et al. demonstrated that hippocampus-specific BDNF deletion impairs novel object recognition and spatial learning by the Morris water maze test [50]. In our previous report, we also reported hippocampus-dependent behavioral abnormalities due to reduced Bdnf expression caused by DNA hypomethylation during adolescence [30]. Considering these findings, although additional experiments are necessary to validate, it is likely that behavioral abnormalities are also induced in the present model.

We found that excessive HFCS solution intake during Period I increased the DNA methylation rate of the CRE-binding region in the *Bdnf* gene (Fig 4). The CRE-binding region is a binding site for CREB, a transcription factor that plays an important role in the promoter activity of *Bdnf*. Specific hypermethylation of this region has been reported to result in decreased transcriptional activity [33]. Our previous study analyzed the effect of DNA methylation of the *Bdnf* promoter on its transcriptional activity using a luciferase assay, suggesting that DNA methylation in the CRE-binding region may play important roles in its transcriptional activity [30]. Taken together with reports that a few percent change in the promoter methylation level of Bdnf can affect its transcription, DNA hypermethylation of CREB binding sites observed in this study may lead to transcriptional repression of the *Bdnf* gene [52].

In this study, a 20% HFCS solution was used. Twenty percent HFCS solution has a concentration comparable to that of a typical soft drink and contains approximately 11% fructose. This is approximately 20% of the total daily caloric intake derived from fructose, which is comparable to the intake of the top 5% of fructose users in the US [53]. Therefore, this research model seems to be relatively consistent with human lifestyle. In this context, we observed that fructose intake during Period I has a particularly strong negative impact on the hippocampus and considering the high fructose intake in 12–18-year-olds, excessive fructose intake during Period I should be avoided.

This study has several limitations. First, this study used an ad libitum model. The calories derived from fructose out of total calories were similar between periods. While, HFCS intake differed with growth; future studies are needed to examine the effects of a controlled HFCS intake. Second, it is unclear whether fructose acts directly on the hippocampus or indirectly through its metabolites. Fructose is absorbed from the small intestine and transported to the liver, where it is rapidly metabolized by phosphofructokinase, a rate-limiting enzyme located upstream of the glycolytic system. Therefore, the effects of the metabolites cannot be ruled out. As reported by Bouvet et al., fructose appeared to be imported into the rat brain by CT imaging [54]. In addition, the presence of glucose transporter 5, which plays a role in fructose transport, has been reported. Based on these findings, we hypothesized that fructose absorbed from the small intestine by oral administration was directly taken up by the hippocampus and affected the expression level of Bdnf [55, 56].

## Conclusions

In this study, we found that fructose intake decreased Bdnf expression in the hippocampus only during childhood and adolescence but not in adulthood. This was also found to be due to modifications in DNA methylation modification. This study provides an insight into the negative effects of excessive HFCS intake in children.

## Supporting information

S1 Table All data for gene expression analysis. The table shows the Ct value determined by the real-time PCR method.
(PDF)

S1 Fig Gel images for western blotting analysis are shown. The membranes were cut according to the molecular weight of the target protein.
(PDF)
