# Supplementary material for: Differential effects of excess high-fructose corn syrup on the DNA methylation of hippocampal neurotrophic factor in childhood and adolescence
Source: PLoS One. 2022 Jun 17;17(6):e0270144. doi: 10.1371/journal.pone.0270144 (PMC9205497; doi:10.1371/journal.pone.0270144)
Supplement: S1 Table — The table shows the Ct value determined by the real-time PCR method. (PDF) [file pone.0270144.s001.pdf]

Period I

Group	Ct value (Left: Housekeeping Gene, Right: Target Gene)							
	Actb	Bdnf Ⅳ	Actb	Ngf	Actb	Nt3	Actb	Nt4
Control	20.38	25.32	20.38	23.99	20.38	26.31	20.38	32.69
	20.51	26.29	20.51	24.20	20.51	26.87	20.51	32.28
	19.60	24.43	19.60	23.11	19.60	25.65	19.60	31.63
	19.13	24.20	19.13	23.13	19.13	25.85	19.13	32.24
	19.38	24.84	19.38	23.62	19.38	25.48	19.38	33.50
HFCS	23.35	29.10	23.35	25.94	23.35	28.57	23.35	35.12
	21.36	27.45	21.36	25.43	21.36	27.79	21.36	33.23
	19.72	25.44	19.72	23.47	19.72	25.12	19.72	33.75
	20.16	25.71	20.16	23.63	20.16	25.48	20.16	34.10
	19.96	25.57	19.96	23.97	19.96	26.57	19.96	30.78
	22.58	28.55	22.58	26.15	22.58	29.00	22.58	33.75

Period II

Group	Ct value (Left: Housekeeping Gene, Right: Target Gene)							
	Actb	Bdnf Ⅳ	Actb	Ngf	Actb	Nt3	Actb	Nt4
Control	18.76	23.60	15.83	25.63	15.83	25.05	15.83	32.37
	19.18	23.72	16.12	25.97	16.12	25.86	16.12	32.03
	19.18	23.71	16.26	26.30	16.26	26.29	16.26	32.07
	19.22	24.09	16.67	26.64	16.67	26.28	16.67	32.46
	19.08	24.11	15.93	26.11	15.93	26.46	15.93	32.08
	18.94	24.11	15.98	25.78	15.98	25.10	15.98	32.34
	18.94	24.31	15.92	26.08	15.92	25.06	15.92	32.33
	19.27	24.66	16.24	26.23	16.24	25.58	16.24	32.57
HFCS	18.72	23.94	15.80	25.96	15.80	24.85	15.80	31.89
	19.22	24.10	16.39	26.43	16.39	26.57	16.39	31.90
	19.00	23.83	15.97	25.98	15.97	25.17	15.97	31.82
	18.71	23.51	16.04	25.86	16.04	25.47	16.04	32.23
	18.62	23.79	16.12	25.82	16.12	25.00	16.12	31.54
	18.80	24.08	16.06	25.76	16.06	25.25	16.06	32.36
	18.84	23.73	15.89	26.08	15.89	26.05	15.89	31.36

Period III

Group	Ct value (Left: Housekeeping Gene, Right: Target Gene)							
	Actb	Bdnf Ⅳ	Actb	Ngf	Actb	Nt3	Actb	Nt4
Control	19.50	23.81	19.50	26.36	19.50	26.51	19.50	32.46
	20.11	24.12	20.11	27.36	20.11	27.05	20.11	31.69
	19.93	23.66	19.93	26.53	19.93	25.70	19.93	31.62
	19.38	23.10	19.38	26.31	19.38	26.27	19.38	31.89
	19.85	22.98	19.85	25.90	19.85	25.91	19.85	31.86
	19.40	22.92	19.40	25.95	19.40	25.61	19.40	31.39
	19.25	23.10	19.25	26.14	19.25	26.40	19.25	31.49
HFCS	19.71	22.51	19.71	25.71	19.71	25.74	19.71	30.91
	19.42	22.53	19.42	25.82	19.42	25.78	19.42	31.86
	20.26	24.11	20.26	26.49	20.26	26.53	20.26	33.08
	19.24	23.42	19.24	26.12	19.24	25.69	19.24	32.05
	19.37	23.30	19.37	26.02	19.37	25.71	19.37	32.36
	19.66	22.50	19.66	25.94	19.66	26.23	19.66	30.62
