# Supplementary figures and images for: Differential effects of excess high-fructose corn syrup on the DNA methylation of hippocampal neurotrophic factor in childhood and adolescence
Source: PLoS One. 2022 Jun 17;17(6):e0270144. doi: 10.1371/journal.pone.0270144 (PMC9205497; doi:10.1371/journal.pone.0270144)

# Period I

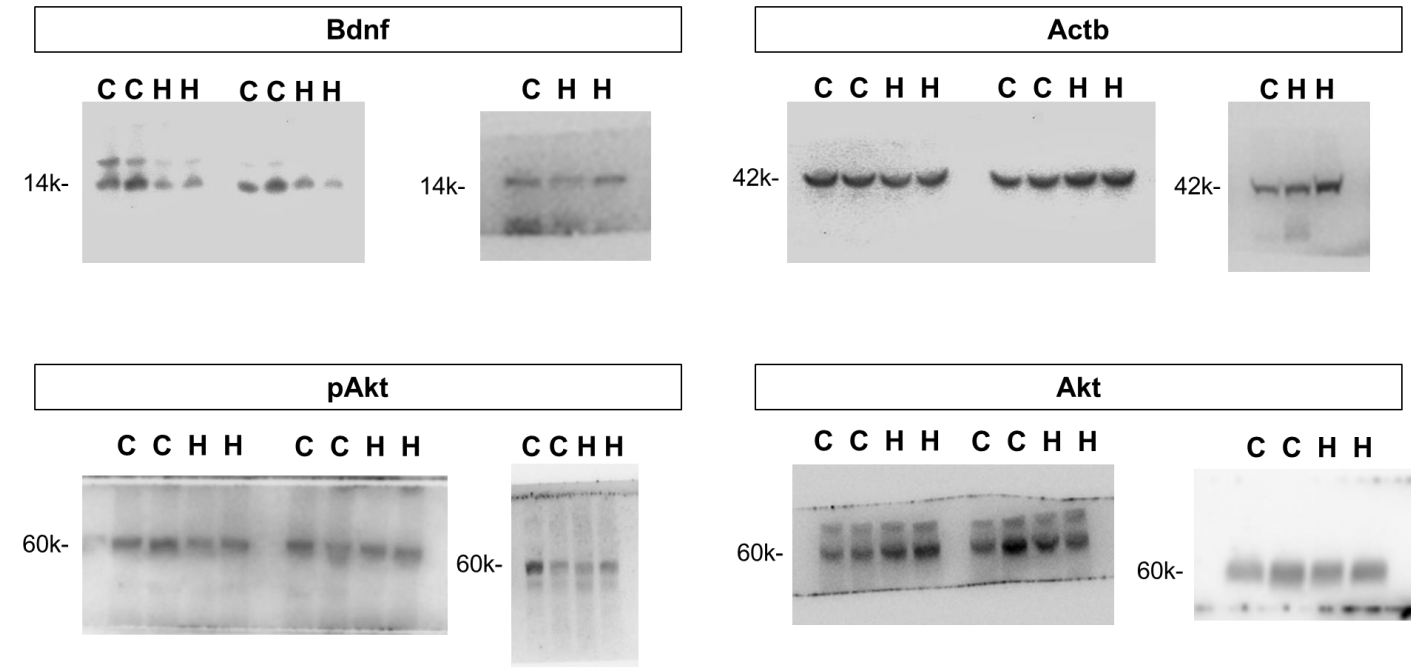

# Period II

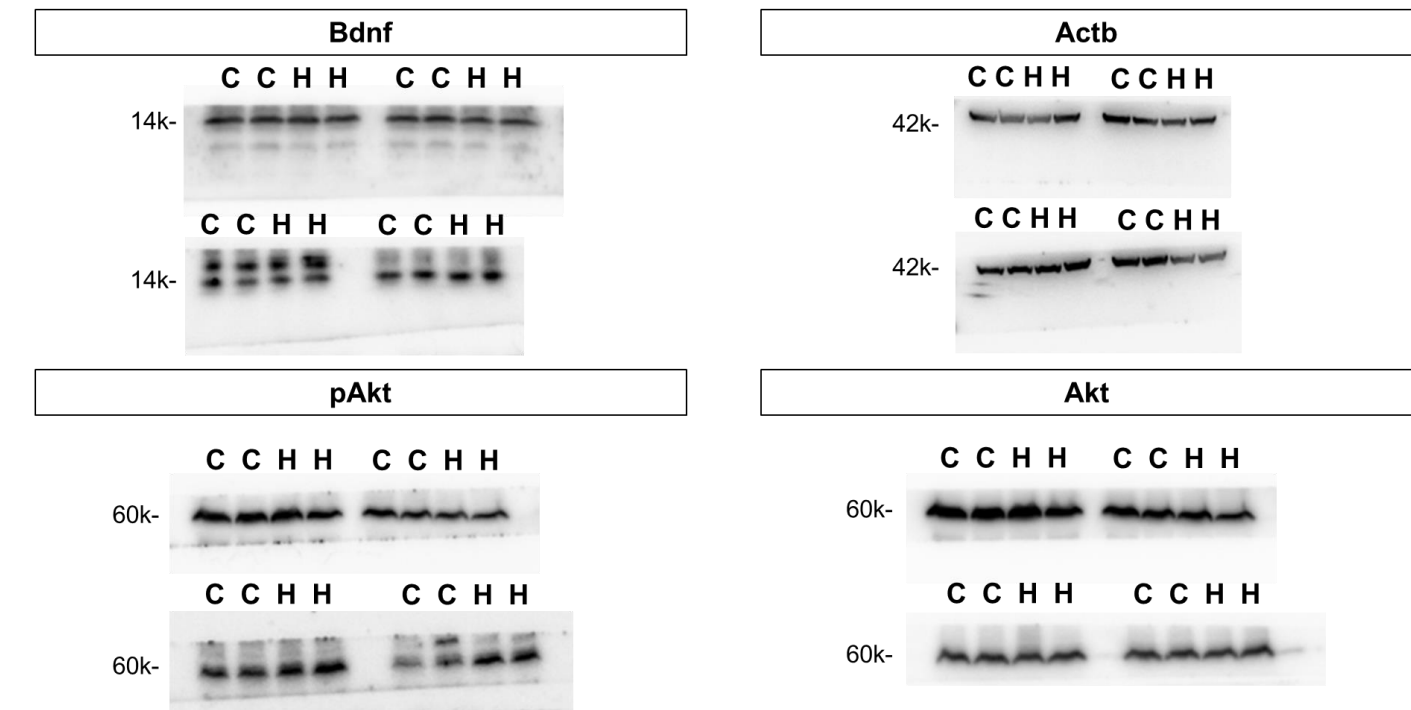

# Period III

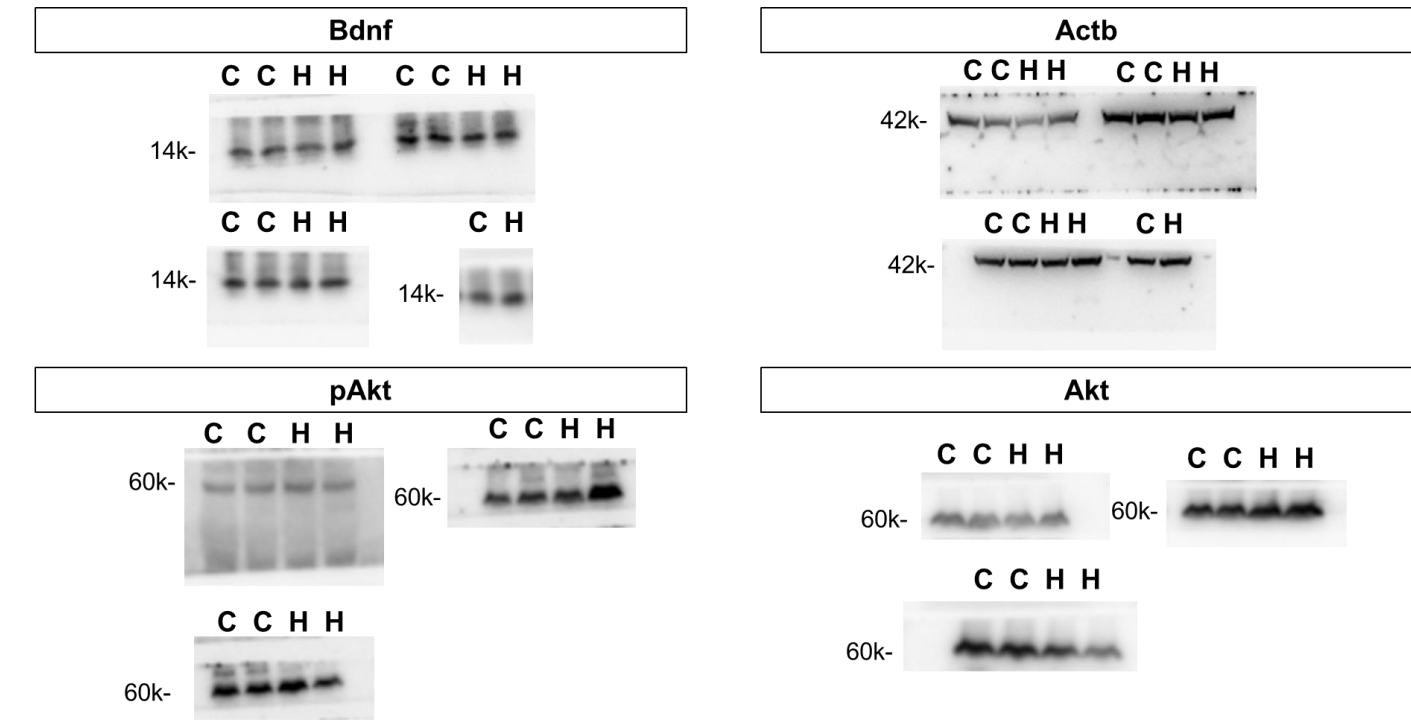

Supplement: S1 Fig — The membranes were cut according to the molecular weight of the target protein. (PDF) [file pone.0270144.s002.pdf]
